# Interplay among *Pseudomonas syringae* HrpR, HrpS and HrpV proteins for regulation of the type III secretion system

**DOI:** 10.1111/1574-6968.12476

**Published:** 2014-06-19

**Authors:** Milija Jovanovic, Edward Lawton, Jörg Schumacher

**Affiliations:** Department of Life Sciences, Imperial College LondonLondon, UK

**Keywords:** σ^54^, AAA+, bEBPs, PspF, RNAP

## Abstract

*Pseudomonas syringae* pv. *tomato* DC3000, a plant pathogenic gram-negative bacterium, employs the type III secretion system (T3SS) to cause disease in tomato and *Arabidopsis* and to induce the hypersensitive response in nonhost plants. The expression of T3SS is regulated by the HrpL extracytoplasmic sigma factor. Expression of HrpL is controlled by transcriptional activators HrpR and HrpS and negative regulator HrpV. In this study, we analysed the organization of HrpRS and HrpV regulatory proteins and interplay between them. We identified one key residue I26 in HrpS required for repression by HrpV. Substitution of I26 in HrpS abolishes its interaction with HrpV and impairs interactions between HrpS and HrpR and the self-association of HrpS. We show that HrpS self-associates and can associate simultaneously with HrpR and HrpV. We now propose that HrpS has a central role in the assembly of the regulatory HrpRSV complex. Deletion analysis of HrpR and HrpS proteins showed that C-terminal parts of HrpR and HrpS confer determinants indispensable for their self-assembly.

## Introduction

*Pseudomonas syringae* pv. *tomato* DC3000 is a plant pathogen that infects tomato (*Solanum lycopersicum*) and *Arabidopsis thaliana*. The *hrp* gene cluster encodes a type III secretion system (T3SS) through which bacteria deliver effector proteins into the plant inducing disease symptoms in host or the hypersensitive response in nonhost plants. Activation of the *hrp* regulon is dependent upon three proteins, HrpL, HrpR and HrpS (Hutcheson *et al*., 2001; Jovanovic *et al*., 2011). HrpL is an alternative sigma factor of the extracytoplasmic factor family that activates expression of other *hrp* and avirulent genes. Transcription of *hrpL* is controlled from the σ^54^-dependent *hrpL* promoter that requires HrpR and HrpS for activity (Hutcheson *et al*., 2001; Ortiz-Martin *et al*., 2010a). The negative regulator HrpV acts upstream of the HrpRS via HrpV-HrpS proteins interactions (Preston *et al*., 1998; Wei *et al*., 2005; Ortiz-Martin *et al*., 2010b).

HrpR and HrpS proteins are bacterial enhancer-binding proteins (bEBPs) that operate as a highly co-dependent hetero-hexameric complex (Jovanovic *et al*., 2011). bEBPs are AAA+ proteins adopting distinct functional subunit states within hexameric assemblies to bind and hydrolyse ATP. The particular nucleotide-dependent conformations of individual subunits allow the binding to, movement of and dissociation from σ^54^ to cause the ATPase-dependent open promoter complex formation. Most of the bEBPs contain three functional domains: (1) N-terminal regulatory domain that controls the self-association of the transcriptional activator in response to a particular signal, (2) conserved AAA+ (central) domain responsible for interaction with σ^54^ and ATP binding and hydrolysis and (3) C-terminal DNA-binding domain that contains a characteristic helix-turn-helix (HTH) DNA-binding structure (Ninfa *et al*., 1987; Wedel *et al*., 1990; Dworkin *et al*., 1997; Schumacher *et al*., 2006; Jolly *et al*., 2010; Joly *et al*., 2012). HrpR and HrpS do not contain the N-terminal regulatory domain and instead are controlled by their negative regulator HrpV acting *in trans* (Wei *et al*., 2005; Jovanovic *et al*., 2011).

**Fig. 1:**
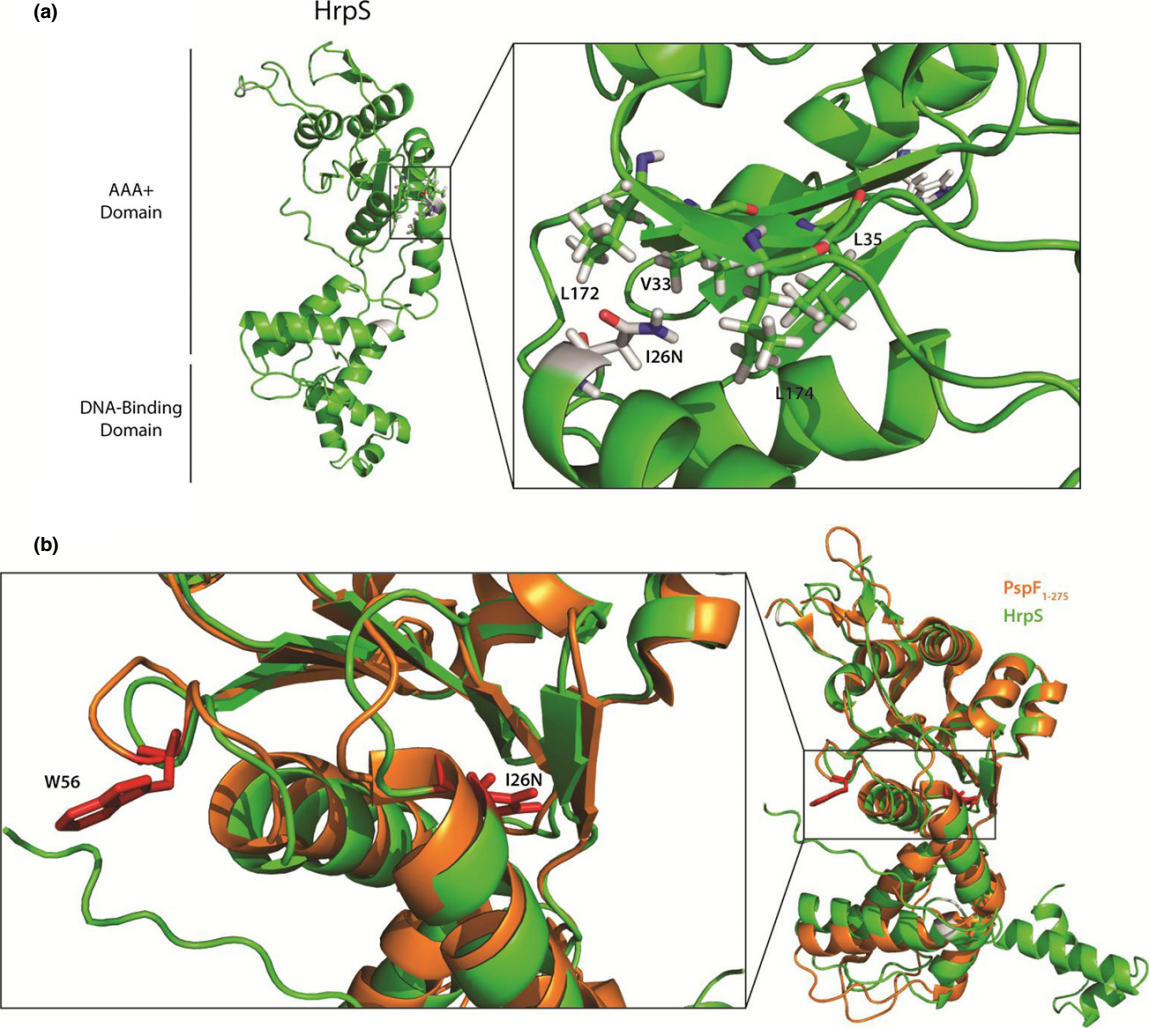
Structural model of HrpS carrying substitution I26N. (a) A predicted structural model of HrpS generated by I-TASSER (Zhang, 2008) shows that the mutant I26N is surface-exposed, where I26 would have originally been part of a strong hydrophobic patch predicted to be disrupted by asparagine substitution. (b) PspF is a homomeric homologue of HrpS and is negatively regulated by *in trans* acting PspA, dependent upon the surface-exposed residue W56 (Rappas *et al*., 2005). I26 and W56 are not located in the same position suggesting different mechanism of HrpS-negative regulation by HrpV.

Heteromeric AAA+ proteins are relatively rare in prokaryotes; the only known bEBPs that form a hetero-oligomer are HrpR and HrpS from *P. syringae* and FleQ and FleT from *Rhodobacter sphaeroides* (Poggio *et al*., 2005). Our results based on a three-component bacterial two hybrid (BACTH) assay showed that the presence of HrpV strongly influences the HrpR–HrpS interaction and HrpS self-association (Jovanovic *et al*., 2011). How HrpV might interact with HrpS within the HrpRS complex to regulate T3SS gene expression is largely unknown, and no obvious analogous bEBP systems offer detailed inferential insights. For example, PspA binds a patch of hydrophobic amino acids in PspF for repression (Zhang *et al*., 2013), but such a patch appears not to be present in HrpS for potential HrpV binding (Fig. 1 and Supporting Information, Fig. S1). Here, we identified and characterized the HrpS residues (I26/R90/R185/H251) important for the repression of HrpS by HrpV. We also deduced the self-associated state of HrpS bound by HrpV and the determinants for this self-association. *In vivo* analyses show that mutation I26N impairs interaction between HrpS and HrpR and HrpS self-association and abolishes HrpS–HrpV interaction. We now suggest a role for HrpS as the key central component in the assembly of a HrpR,S,V regulatory hub and show that important oligomerization determinants lie in the C-terminus of HrpR and HrpS proteins.

## Materials and methods

### Bacterial strains, plasmids and growth conditions

Bacterial strains and plasmids used are described in Table S1. Antibiotics were ampicillin 100 μg mL^−1^; kanamycin 25 μg mL^−1^, for BACTH assay 50 μg mL^−1^, and chloramphenicol 30 μg mL^−1^. *Escherichia coli* strains were grown in Luria–Bertani (LB) medium at 25 °C.

### Recombinant DNA techniques

For plasmid DNA purification was used Mini column plasmid purification kit (Qiagen). Restriction enzymes were used according to the manufacturer's instructions.

### Plasmid constructions for BACTH system

To construct plasmids used in the BACTH assay, the genes encoding Hrp proteins or their variants were amplified by PCR; PCR fragments were digested with XbaI and KpnI and cloned into the XbaI/KpnI-digested pUT18C and pKT25 vectors. For three-component BACTH system, we used plasmids co-expressing hybrid T18C-/T25-Hrp protein(s) and WT HrpS (or its variants) or HrpV proteins. Genes encoding the WT HrpS, HrpS(I26N), HrpS(K233D) and HrpV proteins were amplified by PCR (restriction site KpnI and ribosome-binding site and/EcoRI were included in the PCR primers used for amplification), and obtained PCR fragments were cloned into the KpnI/EcoRI-digested plasmid pUT18C-*hrpV* and pUT18C-*hrpS*, or into KpnI-digested pUT18C-*hrpR plasmid*. All plasmid constructs were verified by sequencing.

### Error-prone PCR mutagenesis of HrpR and HrpS

Error-prone PCR was performed with 800 ng (for 1–4.5 mutations kb^−1^) of pUC18-*hrpRS* using GeneMorph II Random Mutagenesis Kit according to the manufacturer's instructions.

### Construction and screening of mutant library

The amplified *hrpRS* PCR products were recovered by QIAquick gel extraction kit (Qiagen), digested with XbaI and KpnI and cloned into the corresponding restriction sites of vector pAPT110, and *E. coli* XL10 gold cells (Stratagene) were transformed. Transformation mix was plated onto Kan25 selective LA plates, and transformants were collected and transferred into 100 mL LB medium containing 25 μg mL^−1^ Kan. After overnight growth at 37 °C, plasmid DNA was extracted by QIAquick midi plasmid purification kit, MC4100 *hrpL*::*lacZ*/pBAD*hrpV* reporter strain was then transformed and transformation mix collected and plated on LB (Luria–Bertani) plates supplemented with 25 μg mL^−1^ kanamycin, 30 μg mL^−1^ chloramphenicol, 80 μg mL^−1^ X-gal (5-bromo-4-chloroindol-3-yl β-d-galactopyranoside), 0.5 mM IPTG (isopropyl-β-d-thiogalactoside) and 0.2% arabinose and incubated at 25 °C for 4–5 days. Blue colonies were saved for further plasmid isolation and DNA sequence analysis to identify the plasmids containing mutations in *hrpR* and *hrpS* genes. Among *c.* 10 000 colonies screened, we identified three different mutants: HrpS(I26N), HrpS(R185H) and HrpS(R90H/H251Q).

### BACTH assay

BACTH assay was performed as described in Fig. 3.

### β-Galactosidase assay

β-Galactosidase assay was performed as described by Miller, 1971. Briefly, for β-Gal measurements, cells (0.5–0.8 mL of O/N culture) were permeabilized by vortexing for 10 s in Z buffer (0.2–0.5 mL), 60 μL chloroform and 30 μL 0.1% SDS and incubated at 28 °C for 6 min. The reaction was started by adding 0.2 mL of 4 mg mL^−1^ ONPG; after development of yellow colour, the reaction was stopped by adding 0.5 mL of 1 M Na_2_CO_3,_ and incubation time was recorded. The β-Gal activity was calculated according to the equation: 1000 × OD_420_ − (1.75 × OD_550_)/incubation time (min) × vol. of cells (mL). Three to fourfold higher level than level measured for BTH101 (pUT18C/pKT25) was considered as interaction.

### Western blots

For Western blotting, cell pellets were resuspended in Laemmli loading buffer (Sigma-Aldrich) and run on 12.5% SDS-PAGE. Proteins were transferred to PDVF transfer membrane (Millipore Cor.) by electroblot, and membranes were exposed first to primary antibodies HrpR, HrpS or HrpV and detected with anti-rabbit monoclonal antibodies conjugated with horse radish peroxidise (GE Healthcare) and Pierce ECL Western blotting substrate (Thermo Scientific).

## Results

### HrpS I26 residue is necessary for HrpV repression

We reported that HrpV binds HrpS but not HrpR and that this binding represses HrpRS activity (Jovanovic *et al*., 2011). To identify residues or motifs in HrpS important for its interaction with HrpV and to understand the basis of HrpV repression, we performed random PCR mutagenesis to potentially obtain the mutants within HrpRS complex resistant to HrpV negative control (see Materials and methods). We detected three HrpS variants, I25N, R185H and R90H/H251Q, enabling HrpRS complex (containing the HrpS mutant) to escape the repression by HrpV (Figs 1 and S1). Identified mutant residues in HrpS were also substituted with alanine to remove their side chains to distinguish if their escape phenotypes were side chain specific.

**Fig. 2:**
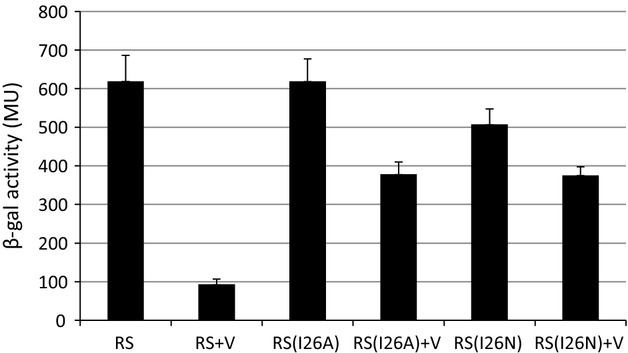
Transcription activity of HrpS and HrpS variants (HrpSI26A, HrpSI26N) *in vivo*, within HrpRS complex that escapes negative regulation by HrpV. HrpRS proteins or their variants were co-expressed from pAPT110 vector; HrpV was expressed from pBAD-based vector. Reporter strain MJ2806 carrying chromosomal *hrpL::lacZ* fusion and HrpRS and HrpV constructs was grown in LB liquid culture supplemented with 25 μg mL^−1^ Kan and 30 μg mL^−1^ Cm; overnight culture was diluted 50 times, grown at 25 °C until OD_600_ 0.4–0.6, induced with 0.5 mM IPTG (HrpRS) and 0.2% arabinose (HrpV) and grown for next 3 h. Activity of *hrpL::lacZ* promoter fusion was measured using β-Galactosidase assay. Each bar represents the mean value with standard deviations of results obtained from three independent biological samples.

All mutants were tested for *in vivo* transcription activity of the HrpRS complex, and Figs 2 and S2 show the effect of selected mutations and their alanine variants on the ability of the HrpRS complex to activate transcription from the *hrpL* promoter with or without HrpV. Results showed that (1) substitution I26N in HrpS drastically changes sensitivity of the HrpRS complex to HrpV, (2) substitution R90A/H has a negative effect on HrpRS transcription activation capacity while sensitivity to HrpV remains similar to that obtained with WT HrpRS and (3) substitutions R185A/H and H251A/Q did not affect HrpRS activity but decreased repression by HrpV up to twofold compared with WT HrpRS.

**Fig. 3:**
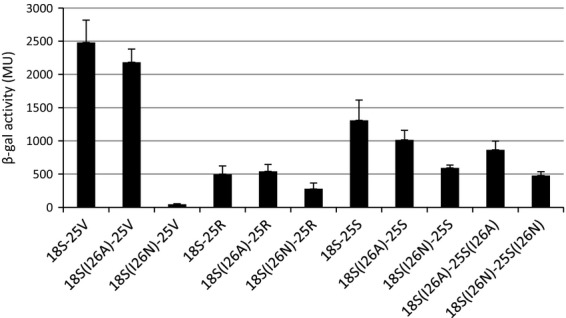
Interactions between the HrpS(I26N) variant and HrpV, HrpR and HrpS proteins detected by BACTH system. The BACTH system is based on the reconstruction of adenylate cyclase (AC) enzymatic activity. Genes encoding proteins of interest are cloned into pKT25 and/or pUT18C plasmids (carrying the N-terminal (T25) and C-terminal (T18) fragments of *Bordatela pertussis* AC) creating gene fusions with T18 or T25 complementary fragments of AC. If proteins of interest interact, T18 and T25 fragments are in close proximity and reconstitute functional AC and consequently to synthesis of cAMP that together with CAP stimulates transcription of the chromosomal *lacZ* reporter gene. The efficiency of complementation between two hybrid proteins was estimated using the β-Galactosidase assay. Recombinant pUT18C and pKT25 carrying the *hrp* genes of interest (where N-terminus of protein is fused with T18 or T25 AC fragments) were used to co-transform *Escherichia coli* BTH101 strain; cells were plated on the selective media: LB supplemented with 100 μg mL^−1^ of Amp, 50 μg mL^−1^ of Kan, 0.5 mM IPTG and X-Gal and grew for 2 days at 25 °C. Transformants were grown in LB broth in the presence of 0.5 mM IPTG and appropriate antibiotics at 25 °C for 20–22 h, and efficiency of interaction was measured by β-Galactosidase assay. Of 18 and 25 stand for T18 and T25 AC fragments on pUT18C and pKT25 vector. HrpRS labelled as RS, HrpSI26 substitutions are given in the brackets HrpSI26A (I26A), and HrpSI26N (I26N), HrpV labelled as V. The positive control with constructs expressing T18-zip and T25-zip yielded 2557 ± 589, while negative control with empty vectors pUT18C and pKT25 yielded 42 ± 9. Each bar represents the mean value with standard deviations of results obtained from three independent biological samples.

To characterize the associations between Hrp proteins and to assess the effect of the changed residues in HrpS for self-association and interaction with HrpV as well as with HrpR, we used the BACTH system (Figs 3 and S3; Karimova *et al*., 1998). BACTH system has been used to characterize the interactions between proteins. It relies on the reconstitution of *Bordatela pertussis* adenylate cyclase (AC) activity where proteins of interest are fused to two fragments T18 and T25 from AC. T18 and T25 fragments are inactive when co-expressed separately but if proteins of interest fused with these fragments interact, then T18 and T25 are brought into close proximity to allow functional complementation, leading to cAMP synthesis and transcriptional activation of the lactose operon. The HrpS, HrpR and HrpV proteins were expressed from pUT18C and pKT25 vectors as hybrid proteins in which the examined protein was fused at the C-terminus of T18 or T25 fragment. Intensity of interactions between the T18 and T25-Hrp hybrid proteins was determined in strain lacking AC by measuring expression of chromosomal *lacZ* using β-galactosidase assay (see Materials and methods). Western blotting data verified (Fig. S4) that any failure to interact with HrpV, HrpR or HrpS (or variants thereof) was not due to protein instability.

Taken together, our results (Figs 3 and S3a–c) showed that (1) alanine substitutions did not change the efficiency of interaction between HrpS and HrpV in any of the HrpS mutants while the substitution I26N abolished the interaction between HrpS and HrpV, other mutations obtained by random PCR mutagenesis did not significantly changed the interaction between HrpS and HrpV, (2) interactions with HrpR were not changed except for HrpS (I26N) variant where the interaction is decreased and (3) substitutions in HrpS(I26N) and HrpS(R185A) impair interaction with WT HrpS and self-association.

We used the crystal structure of the AAA+ domain of the bEBP PspF to model HrpS and so determine the predicted location of the studied residues (Figs 1 and S1). Data obtained from a model HrpS structure based in structural data of a PspF protein showed that HrpS(I26N) is located in a helix 1 of the AAA+ domain. As both I26N and I26A mutations diminish repression by HrpV, our data suggest that residue I26 in HrpS can be directly implicated in transfer of the repression signal received through binding of the HrpV to HrpS and communicated to the HrpR–HrpS interface.

### HrpS can simultaneously bind T18/T25-HrpR and T18/T25-HrpV hybrid proteins

**Fig. 4:**
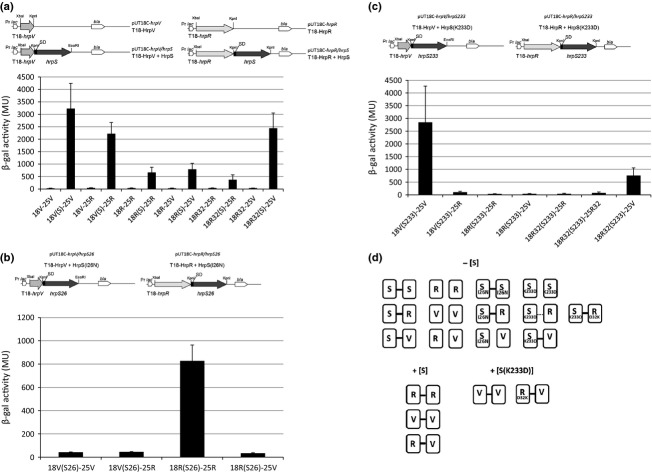
Associations among HrpV and HrpR hybrid proteins in the presence of WT HrpS and different HrpS variants detected by three-component BACTH system. For this experiment, a derivative of pUT18C*hrpR* or pUT18C*hrpV* was constructed that together with hybrid proteins 18-HrpR or 18-HrpV also expresses WT HrpS or one of the HrpS variants I26N or K233D. Interactions between the hybrid proteins were measured and quantified as described in Fig. 3 legend. Each bar represents the mean value with standard deviations of results obtained from three independent biological samples. (a) Schematic drawing of pUT18C*hrpV* and pUT18C*hrpR* plasmids expressing the T18-HrpV and T18-HrpR fusions, and pUT18C*hrpV*/*hrpS* and pUT18C*hrpR*/*hrpS* plasmids coexpressing the T18-HrpV or T18-HrpR fusion and the WT HrpS protein; Pr *lac*- *lac* promoter, SD- Shine-Dalgarno sequence, *bla*-ampiciline resistance. Interaction between HrpR and HrpV hybrid proteins in the absence or in the presence of co-expressed WT HrpS. HrpS (S) from pUT18C vector is co-expressed with T18-HrpR, 18R(S) or T18-HrpV, 18V(S). (b) Schematic drawing of pUT18C*hrpV*/*hrpS26* and pUT18C*hrpR*/*hrpS26* plasmids coexpressing the T18-HrpV or T18-HrpR fusion and the HrpS(I26N) variant. Interaction between HrpR and HrpV in the presence of HrpS(I26N). HrpS(I26N) (S26) from pUT18C vector is co-expressed with T18-HrpR 18R(S26) or T18-HrpV 18V(S26). (c) Schematic drawing of pUT18C*hrpV*/*hrpS233* and pUT18C*hrpR*/*hrpS233* plasmids coexpressing the T18-HrpV or T18-HrpR fusion and the HrpS(K233D) variant. Interaction between HrpR and HrpV hybrid proteins in the presence of HrpS(K233D). HrpS(K233D) (S233) from pUT18C vector is co-expressed with T18-HrpR 18R(S233), T18-HrpR(D32K) 18R32(S233) or T18-HrpV protein 18V(S233). (d) Schematic presentation of the interactions between HrpS, HrpR, HrpV and their variants obtained in the BACTH; boxes represent hybrid proteins; line between boxes, protein–protein interaction; dashed line, weak protein–protein interaction; separated boxes, no protein–protein interaction. [+S] or [+S(K233D)], presence of co-expressed HrpS or its variants; [−S], without co-expressed HrpS.

Our previous studies on interactions between HrpR, HrpS and HrpV proteins showed that HrpS was able to self-associate and to directly interact with the HrpR or HrpV components of the Hrp regulatory system, while HrpR and HrpV are apparently unable to self-associate or interact with each other (Jovanovic *et al*., 2011). Whether HrpS can simultaneously interact with HrpR and HrpV or the association between them is only sequential and so mutually exclusive is unknown. To explore these possibilities, we studied the effect of co-expressed HrpS on interaction between the HrpV and HrpR hybrid proteins in three-component BACTH system (Karimova *et al*., 2005; Jovanovic *et al*., 2011). Here, a derivative of plasmids pUT18C-HrpV and pUT18C-HrpR were constructed to co-express WT HrpS protein or its variants HrpS(I26N) or HrpS(K233D) (Lawton *et al*., 2014). These constructs were tested for interactions with T25-HrpV or T25-HrpR hybrid proteins. As shown in Fig. 4a, co-expression of HrpS enables an association between T18-HrpR and T25-HrpR, T18-HrpV and T25-HrpV, and T18-HrpR and T25-HrpV. These data suggest that co-expressed HrpS molecules might stabilize the HrpR or HrpV complex by interacting simultaneously with both components. In the next set of experiments, we examined the effect of the co-expressed HrpS(I26N) variant (incapable of interaction with HrpV) on HrpR and HrpV association. As shown in Fig. 4b in the presence of HrpS(I26N), the HrpV hybrid proteins were unable to self-associate or to associate with HrpR while association between T18-HrpR and T25-HrpR proteins was still facilitated. These data indicate that in the presence of HrpS(I26N), HrpV is unable to interact with HrpS and therefore HrpS cannot bridge the two hybrid proteins T18-HrpV and T25-HrpV or T18-HrpV and T25-HrpR. We also analysed association between HrpR and HrpV hybrid proteins when HrpS(K233D) variant was co-expressed (Fig. 4c). We showed previously that HrpR D32 and HrpS K233 residues act as a pair, most likely directly interacting and so directly contributing to the binding interaction between HrpR and HrpS subunits (Lawton *et al*., 2014). Substitution K233D in HrpS abolishes self-association and significantly decreases interaction with WT HrpR with no effect on interaction with HrpV (Fig. 4d). The D32K substitution in HrpR did not alter the interaction with WT HrpS but compensates for a negative effect of HrpS K233D substitution when interacting with HrpR. In the presence of HrpS(K233D), we detected association between two HrpV hybrid proteins and between the HrpR(D32K) variant and HrpV protein (Fig. 4c). We did not detect interaction between two hybrid HrpR proteins or HrpR and HrpV. Surprisingly, we did not detect association between two hybrid HrpR(D32K) variants, whereas we showed that this variant can interact with HrpS(K233D). Presence of HrpS(K233D) did not promote an association between hybrid HrpR(D32K) and HrpV proteins. Taken together, these data suggest that (1) HrpS has distinct sites of interaction, one for HrpV and one for HrpR, (2) inability of HrpS(K233D) to self-associate could abolish association (bridging) of two hybrid HrpR(D32K) in the three-component BACTH system and (3) the presence of HrpV might affect self-association of HrpS(K233D) variant. One question to be answered is the lack of association between the two hybrid HrpR(D32K) variants in the presence of co-expressed HrpS(K233D).

### Effects of HrpV on HrpS self-association

**Fig. 5:**
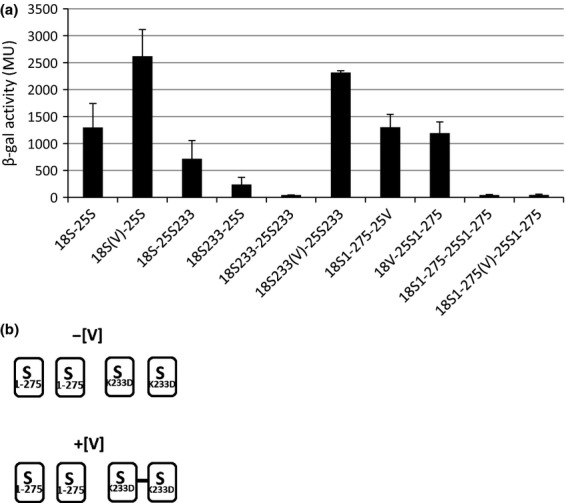
(a) Effects of co-expressed HrpV on self-association of HrpS(K233D) and HrpS_1–275_ variants. For this experiment, a derivative of pUT18C*hrpS* or its variants was constructed that together with hybrid proteins 18-HrpS or 18-HrpS(K233D) or HrpS_1–275_ also expresses HrpV (V). Interactions between the hybrid proteins were measured and quantified as described in Fig. 3 legend. Each bar represents the mean value with standard deviations of results obtained from three independent biological samples. (b) Schematic presentation of the interactions between HrpS variants. Boxes represent hybrid proteins; line between boxes, protein–protein interaction; separated boxes, no protein–protein interaction. [+V], presence of co-expressed HrpV; [−V], without co-expressed HrpV.

To assess the effects of HrpV on HrpS(K233D) variant, we analysed HrpS self-association for its sensitivity to the presence of HrpV. Using three-component BACTH system, we showed previously that HrpV reinforces the HrpS self-association and interactions between HrpR and HrpS (Jovanovic *et al*., 2011). Therefore, to delineate a potential topological effect of HrpV in these interactions, we used three-component BACTH system to test whether HrpV could stimulate interactions between full-length HrpS(K233D) and the isolated HrpS AAA+ domain (HrpS_1–275_), both defective in dimerization (Fig. 5a and b). The results revealed that HrpV strongly enhances self-association of HrpS(K233D), indicating that action of the HrpV is independent on mutations in HrpS(K233D).

Furthermore, we showed that HrpV could not facilitate the interaction between HrpS_1–275_ hybrid proteins, suggesting that effect of HrpV occurs only with the full-length HrpS protein. This particular result supports the previous observations that HTH domain is an important dimerization determinant in HrpS and other bEBPs (North & Kustu, 1997; Pelton *et al*., 1999). These outcomes also suggest that HrpS to bridge two hybrid proteins should form dimers rather than be a monomer. Wild-type HrpS self-associates but HrpS(K233D) variant is dimerization defective. Hence, when HrpV is present, dimerization of HrpS(K233D) occurs and so the HrpS(K233D) dimer can bridge two hybrid proteins, T18C-HrpR/T25-HrpV or T18CHrpV-T25-HrpV.

### Roles for C-terminal regions of HrpR and HrpS

**Fig. 6:**
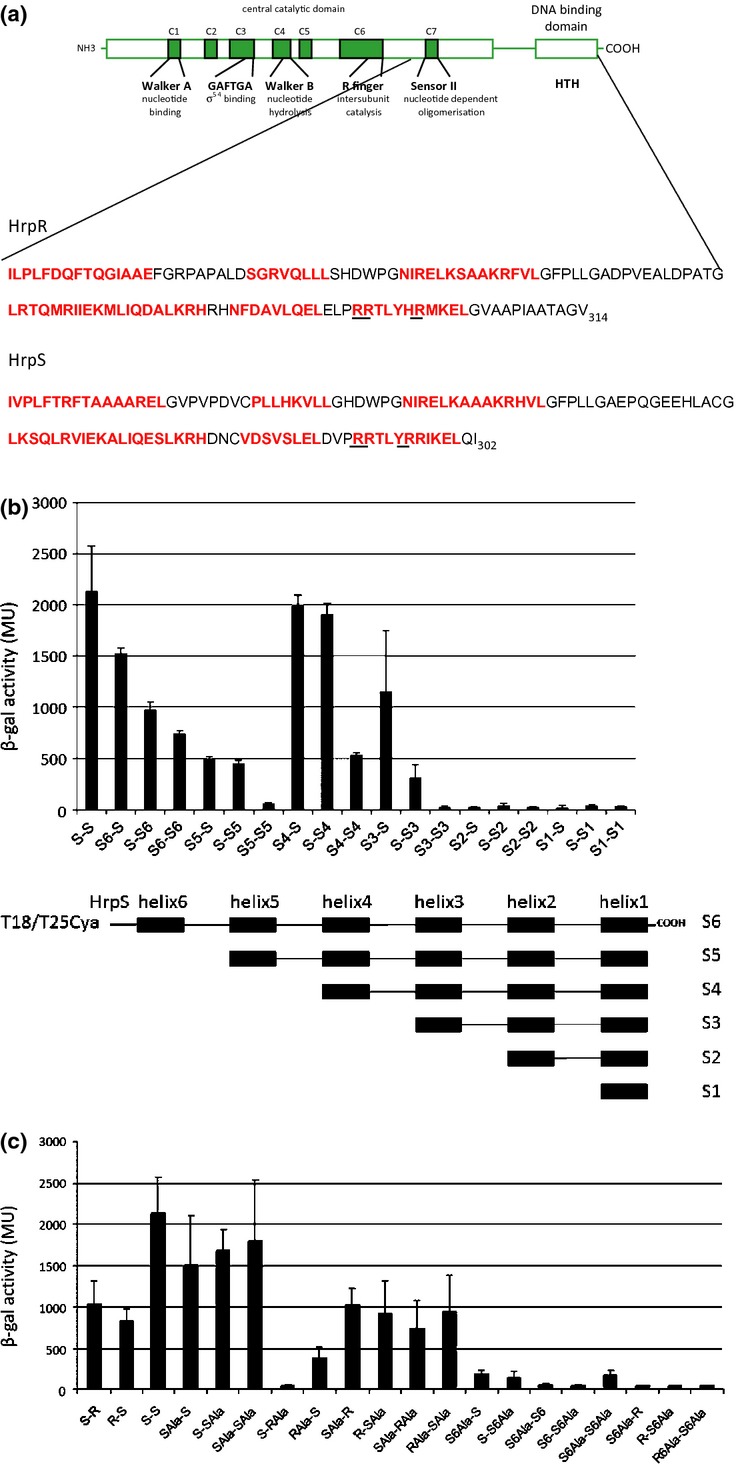
Roles of C-terminal sequences in HrpRS assemblies. (a) Schematic figure represents the HrpR and HrpS domain organizations and the sequence of the six C-terminal helices (red letters). The arginine to alanine substitutions are underlined. (b) Interactions between the HrpS derivatives (S1–S6) and WT HrpS protein. The schematic figure of the HrpS variants containing last 6 C-terminal helices- (S6), 5 helices- (S5), 4 helices- (S4), …and 1 helix- (S1) fused to the T18 or T25 fragment of CA are given below. (c) Interactions between the HrpR R292A/R293A/R298A (RAla) and HrpS R290A/R291A/R295A (SAla) variants. Interactions between the hybrid proteins were measured and quantified as described in Fig. 3 legend. Each bar represents the mean value with standard deviations of results obtained from three independent biological samples.

To expand our understanding of HrpR and HrpS associations in a functional complex and subsequent biological implications *in vivo*, we explored the ability of the HrpR and/or the HrpS C-terminal regions to interact using the BACTH system. Based on the HrpR and HrpS secondary structure predictions, several different constructs were created and the peptides comprising the last six C-terminal α-helices were expressed in BATCH system (e.g. for HrpS – S1, S2, S3, S4, S5, S6; the last two α-helices 1 and 2 present a specific DNA-binding HTH motif; Fig. 6a and b). The ability of each construct to dimerize was compared with the level of interaction between wild-type HrpR and HrpS proteins. We could not detect any of the HrpR-based peptides or their interactions, suggesting that these derivatives are unstable when expressed in a BACTH system. In turn, the results of the BACTH assay revealed that the last four C-terminal helices (S4) of HrpS are involved in HrpS self-association (Fig. 6b) but apparently not in a direct interaction between HrpS and HrpR (data not shown). We could not detect S5–S5 self-association, which was unexpected regarding interactions obtained with the S6–S6 and S4–S4 polypeptides. The HrpS derivative containing the last three α-helices (S3) could not self-associate (did not show any S3–S3 interaction), but the interaction was observed between full-length HrpS and the S3 HrpS peptide (Fig. 6b). These results suggest that for the self-association of two HrpS subunits and for their inclusion in the HrpRS oligomeric complex, different regions of the HrpS protein are employed. Lack of interaction between S5 and S5 helices could be explained in the way that the fifth helix in the S5 fragment may not interact itself, but act as a ‘bridge’ to stabilize the fourth and sixth helices. Although the HrpS peptides analysed might have different conformations compared with one in the full-length protein, we showed their potential to self-associate and associate with full-length HrpS protein. S3–S6 constructs self-associate or associate with full-length HrpS, but we could only detect peptides S5 and S6 by Western Blot indicating that S1–S4 peptides are unstable or some of them cannot be recognized by the antibody. Further, we showed that S6 did not detectably interact with HrpV (data not shown), suggesting that the AAA+ domain of the HrpS is important for the interaction with HrpV.

The studies of *S. typhimurium* NtrC showed that derivate with three arginines in the last recognition helix of the HTH motif substituted with alanines abolished NtrC–DNA interaction *in vitro* (Pelton *et al*., 1999). In the same note, our previous studies showed that three alanine substitutions in the recognition helix of HrpR (R292A/R293A/R298A), HrpR(3Ala) and HrpS (R292A/R293A/R298A), HrpS(3Ala) HTH motifs (see Fig. 6a) abolished the activity of HrpRS complex *in vivo* (Jovanovic *et al*., 2011). To explore in more detail the effect of these alanine substitutions on HrpRS communication, we analysed interactions between HrpR(3Ala) and HrpS(3Ala) derivatives (Fig. 6b). Our results showed that HrpS(3Ala)–WT HrpS interaction is moderately affected, while HrpS(3Ala) self-association and interaction with HrpR were unchanged compared with one seen with the WT HrpS (Fig. 6c). In contrast, the HrpR(3Ala) interactions with the WT HrpS were severely affected, suggesting that arginine residues in HrpR (but not HrpS) might have an important role for establishing a HrpR–HrpS interaction. Possibly, the 3Ala substitutions might have caused structural alterations in the HrpR and HrpS consequently affecting the interaction between HrpR and HrpS proteins, which is required to yield the active oligomeric species.

## Discussion

The HrpRS transcriptional activator is negatively controlled by HrpV. Our studies now provide new insights into this control. We show how residues in HrpS involved in negative regulation by HrpV support an interaction network among HrpR, HrpS and HrpV proteins and identify important hetero and homo-dimerization determinants in HrpR and HrpS that are indispensable for production of the active oligomeric HrpRS species. The HrpS I26N mutation abolishes its binding to HrpV and diminishes negative regulation while I26A substitution affects negative control but not the interaction between HrpS and HrpV. We propose that residue I26 in HrpS is needed to transfer the repressive signal received through its binding of HrpV. All other HrpS variants did not significantly change efficiency of interaction with HrpV, suggesting that mutated residues are not involved in direct contact with HrpV. Three-component BACTH system showed that association between HrpR and HrpV proteins (that do not interact in conventional BACTH assay) is enabled in the presence of the HrpS. HrpS most likely co-localizes HrpV and HrpR hybrid molecules sufficiently to obtain adenylate cyclase activity. Experiments with the co-expressed HrpSI26N variant showed lack of HrpV self-association or HrpV-HrpR association, strongly indicating that HrpS bridges between the two hybrid pairs HrpR and HrpR, HrpV and HrpV and HrpR and HrpV.

Data obtained with HrpS(K233D) variant (Fig. 4d) showed that substitution of HrpS(K233D) abolished association between WT HrpR hybrid proteins but not between two HrpV hybrid proteins and between HrpV and HrpR(D32K) hybrid proteins. The lack of association between two HrpRD32K hybrid proteins indicates that more elements than the *in trans* residue congruences, proposed in Lawton *et al*., 2014, must be involved in the assembly of these proteins. Further experiments where co-expressed HrpV strongly enhances interaction between two HrpSK233D variants indicate that for simultaneous association with HrpR and HrpV hybrid proteins, HrpS might be dimeric rather than monomeric. In the absence of HrpV, HrpS(K233D) fails as a fusion protein to self-associate. Moreover, HrpS(K233D) self-association in the presence of HrpV suggests that HrpV binds to at least two subunits of HrpS.

Using BACTH, we showed that HrpV stimulated both the self-association of HrpS and its interaction with HrpR (Jovanovic *et al*., 2011). Our findings with the self-association-deficient HrpS(K233D), where the presence of HrpV strongly stimulated HrpS(K233D) self-association (Fig. 5), suggested that HrpV could either somehow clamp between two HrpS(K233D) molecules or induce structural changes in HrpS subunits that strengthen their self-association. Because HrpV could also enhance associations between HrpS and HrpR, including those between HrpS and HrpR(D32K), it might be that HrpV binding to HrpS induces conformational changes in the HrpS interface that interacts alternatively with HrpS and HrpR.

In the presence of co-expressed HrpV proteins, the HrpS_1–275_-truncated variants lacking the HTH domain could not self-associate, suggesting that for oligomerization, C-terminal sequences are indispensable. Our studies on the last six C-terminal helices and HrpR (3Ala) and HrpS (3Ala) variants showed that important dimerization determinants for HrpS and HrpR do indeed lie in the C-terminus of these proteins. Furthermore, changes in the HrpR HTH domain had more detrimental effects on the association between HrpR and HrpS compared with the equivalent changes in HrpS, suggesting at least some differential contributions between the HrpR and HrpS HTH domains in the overall structural assembly, although the functional importance of these differences remains unclear.

Collectively our data indicated a novel mode of bEBP trans-regulation that is probably special to the unusual heteromeric nature of HrpRS. In *nonsyringae* phytopathogenic *Pseudomonas*, a single-acting HrpS (HrpV regulated) activates the expression of *hrpL* and the co-dependent *hrpRS* has evolved through a gene duplication event, and we proposed that this elaboration may afford the integration of more signals (Jovanovic *et al*., 2011). Our findings now provide an insight in the mechanistic framework for how distinct signals could be integrated. As HrpV appears to specifically act directly on HrpS only and the Lon protease can specifically degrade HrpR (Bretz *et al*., 2002), such an arrangement could for instance reduce signalling interferences that act *via* HrpV and Lon to improve the fidelity of downstream regulatory responses that depend on HrpRS.

## Supporting Information

**Fig. S1.** The amino acid substitutions HrpS(I26N)/(R90H)/(R185H)/(H251Q) on the structural model of HrpS, based on the AAA+ domain of PspF. **Fig. S2.** Transcription activity *in vivo* of HrpS variants, within HrpRS complexes, that escapes negative regulation by HrpV. **Fig. S3.** Binding interactions between the HrpS variants I26, R90, R185 and H251 fused to T18 AC fragment and assayed in the presence of: (a) HrpV fused to T25 AC fragment, (b) HrpR fused to T25 AC fragment, (c) HrpS fused to T25 AC fragment. **Fig. S4.** Western blotting of the Hrp proteins expressed in the BACTH system recognized by corresponding antibody. **Table S1.** Bacterial strains and plasmids used in this study.
