# Supplementary material for: Interplay among Pseudomonas syringae HrpR, HrpS and HrpV proteins for regulation of the type III secretion system
Source: FEMS Microbiol Lett. 2014 Jun 19;356(2):201–11. doi: 10.1111/1574-6968.12476 (PMC4145663; doi:10.1111/1574-6968.12476)
Supplement: Supplementary file 1 [file fml0356-0201-SD1.pdf]

Supplementary Fig. 1

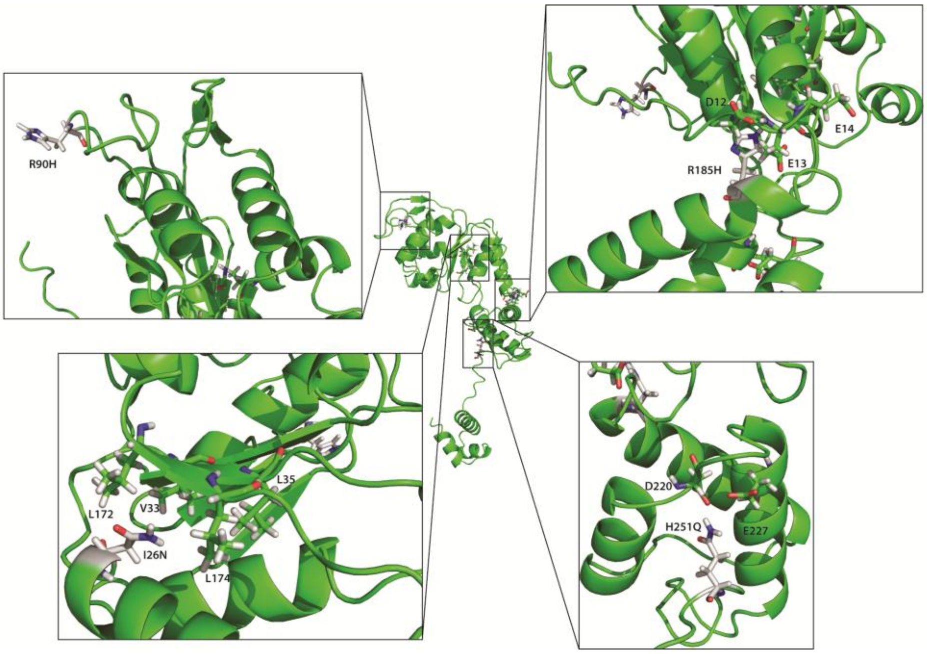

Supplementary Fig. 2

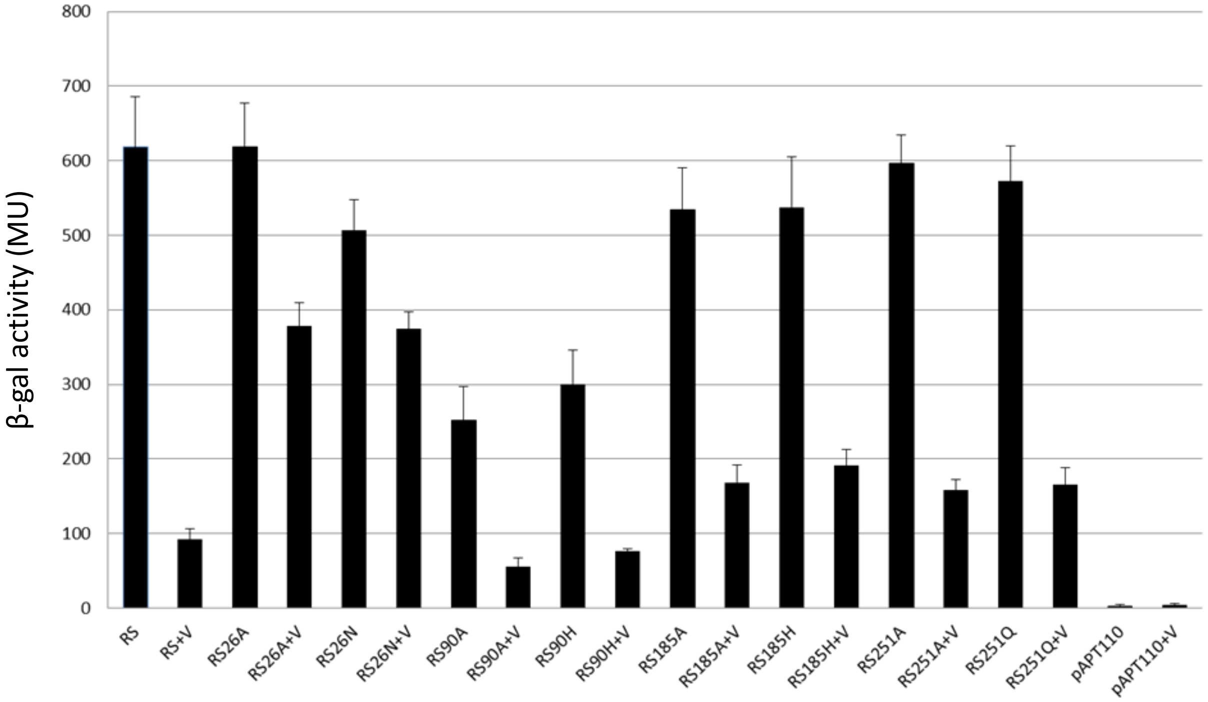

Supplementary Fig. 3

a

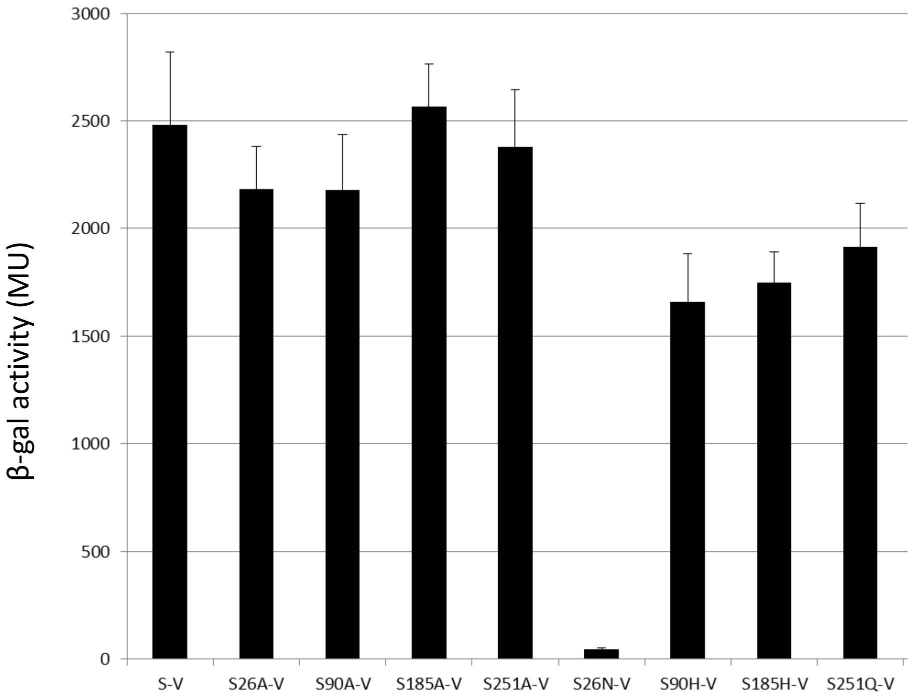

b

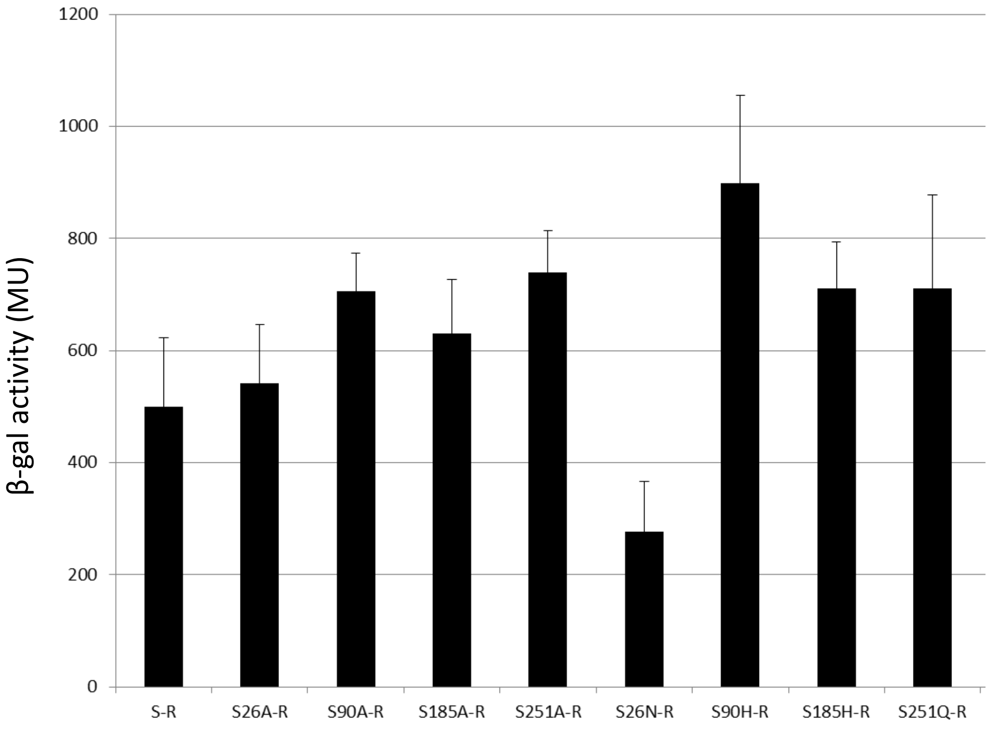

c

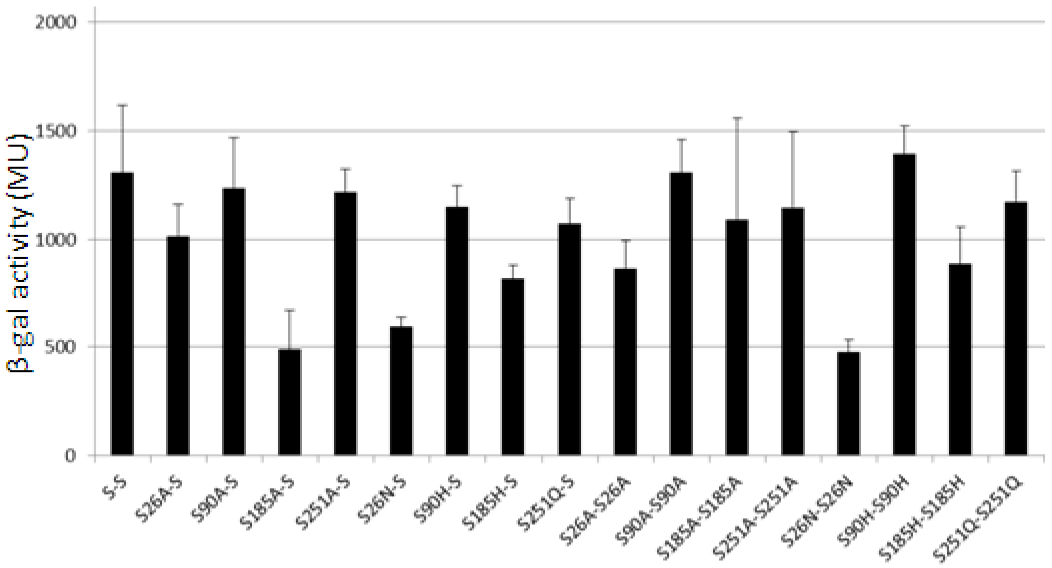

β-gal activity (MU)
2000
1500
1000
500
0
S-S
S26A-S
S90A-S
S185A-S
S251A-S
S26N-S
S90H-S
S185H-S
S251Q-S
S26A-S26A
S90A-S90A
S185A-S185A
S251A-S251A
S26N-S26N
S90H-S90H
S185H-S185H
S251Q-S251Q

Supplementary Fig. 4

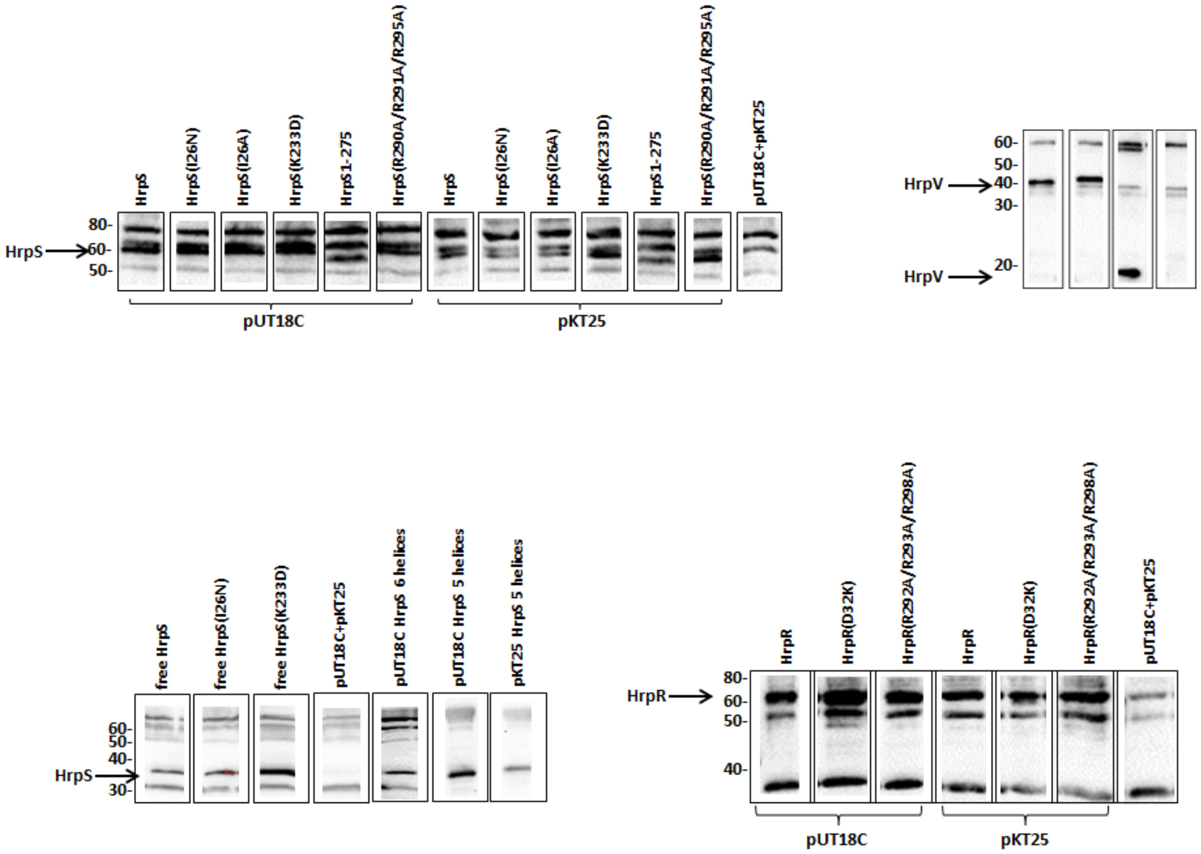

Supplementary Table 1. Bacterial strains and plasmids used in this study.

Strain, vector or plasmid	Genotype or description	Reference or source
MC4100	F' Δ(*argF-lacZYA*)*U169*	Lab. collection
MJ2806	MC4100(*attL hrpL::lacZ bla attR*)	Jovanovic et al., 2011
BTH101	F-, *cya-99, araD139, galE15, galK16, rpsL1 (Str r), hsdR2, mcrA1, mcrB1*	Karimova et al., 1998
pAPT110	Expression vector, Kan^r	Lab. collection
pBAD18	Expression vector, Cm^r	Lab. collection
pUT18C	Contains *B.Pertusis cya* gene T18 fragment,Amp^r	Karimova et al., 1998
pKT25	Contains *B.Pertusis cya* gene T25 fragment, Kan^r	Karimova et al., 1998
pVAM1	pUT18C derivative expressing HrpR	Jovanovic et al., 2011
pVAM21	pUT18C derivative expressing HrpS	Jovanovic et al., 2011
pVAM31	pUT18C derivative expressing HrpV	Jovanovic et al., 2011
pVAM2	pKT25 derivative expressing HrpR	Jovanovic et al., 2011
pVAM12	pKT25 derivative expressing HrpS	Jovanovic et al., 2011
pVAM32	pKT25 derivative expressing HrpV	Jovanovic et al., 2011
pVAM21	pUT18C derivative expressing $HrpS_{1-275}$	Jovanovic et al., 2011
pVAM22	pKT25 derivative expressing $HrpS_{1-275}$	Jovanovic et al., 2011
pMJRS	pAPT110 derivative expressing HrpRS	Jovanovic et al., 2011
pMJV1	pBAD18derivative expressing HrpV	Jovanovic et al., 2011
pVAM21S233D	pVAM21 derivative, expressing HrpS K233D	Lawton et al., 2014
pVAM12S233D	pVAM12 derivative, expressing HrpS K233D	Lawton et al., 2014
pVAM1R32K	p VAM1 derivative, expressing HrpR D32K	Lawton et al., 2014
pVAM2R32K	p VAM2 derivative, expressing HrpRD32K	Lawton et al., 2014
pMJRSI26A	pMJRS derivative, expressing HrpS I26A	This study
pMJRSI26N	pMJRS derivative, expressing HrpS I26N	This study
pMJRSR90A	pMJRS derivative, expressing HrpS R90A	This study
pMJRSR90H	pMJRS derivative, expressing HrpS R90H	This study
pMJRSR185A	pMJRS derivative, expressing HrpS R185A	This study
pMJRSR185H	pMJRS derivative, expressing HrpS R185H	This study
pMJRSH251A	pMJRS derivative, expressing HrpS H251A	This study
pMJRSH251Q	pMJRS derivative, expressing HrpS H251Q	This study
pVAM21S26A	pVAM21 derivative, expressing HrpS S26A	This study
pVAM21S26N	pVAM21 derivative, expressing HrpS S26N	This study
pVAM21R90A	pVAM21 derivative, expressing HrpS R90A	This study
pVAM21R90H	pVAM21 derivative expressing HrpS R90H	This study
pVAM21R185A	pVAM21 derivative expressing HrpS R185A	This study
pVAM21R185H	pVAM21 derivative expressing HrpS R185H	This study
pVAM21H251A	pVAM21 derivative expressing HrpS H251A	This study
pVAM21H251Q	pVAM21 derivative expressing HrpS H251Q	This study
pVAM12S26A	pVAM12 derivative, expressing HrpS S26A	This study
pVAM12S26N	pVAM12 derivative, expressing HrpS S26N	This study
pVAM12R90A	pVAM12 derivative, expressing HrpS R90A	This study
pVAM12R90H	pVAM12 derivative expressing HrpS R90H	This study
pVAM12R185A	pVAM12 derivative expressing HrpS R185A	This study
pVAM12R185H	pVAM12 derivative expressing HrpS R185H	This study
pVAM12H251A	pVAM12 derivative expressing HrpS H251A	This study
pVAM12H251Q	pVAM12 derivative expressing HrpS H251Q	This study

pVAM21S233DV	0.373-kb *KpnI-BamHI* fragment ligated in pVAM21S233D expressing HrpV	This study
pVAM1R32KS233D	0.909-kb *KpnI* fragment ligated in pVAM1R32 expressing HrpSK233D	This study
pVAM21V	0.373-kb *KpnI-BamHI* fragment ligated in pVAM21expressing HrpV	Jovanovic et al., 2011
pVAM31SK233D	0.909-kb *KpnI-BamHI* fragment ligated in pVAM31expressing HrpSK233D	This study
pVAM1SK233D	0.909-kb *KpnI* fragment ligated in pVAM1 expressing HrpSK233D	This study
pVAM31S26N	0.909-kb *KpnI-BamHI* fragment ligated in pVAM31 expressing HrpSI26N	This study
pVAM1S26N	0.909-kb *KpnI* fragment ligated in pVAM1 expressing HrpSI26N	This study
pVAM31S	0.909-kb *KpnI-BamHI* fragment ligated in pVAM31 expressing HrpS	This study
pVAM1S	0.909-kb *KpnI* fragment ligated in pVAM1 expressing HrpS	This study

Figure Legends

Supplementary Fig. 1

The amino acid substitutions HrpS(I26N) /(R90H)/(R185H)/ /(H251Q) on the structural model of HrpS, based on the AAA+ domain of PspF. R90H is located on the L1 loop and may contribute to its exposure. R185 is in close proximity to the negatively charged amino acids D12, E13 and E14 and may have formed an electrostatic interaction that is disrupted by the R185H substitution. I26 is likely to have formed a strong hydrophobic interaction with nearby aliphatic amino acids making the I26N substitution disruptive. H251 is not likely to interact with other amino acids but by introducing the H251Q substitution it is possible that new interactions with D220 and E227 occur.

In the hexameric AAA+ ring assemblies of bEBPs, subunits interact front to back, so that in the heteromeric HrpR-HrpS hexamer minimally one interacting subunit surface of HrpS must be competent to interact with HrpR and HrpS. Because HrpV stimulates both HrpS-HrpS and HrpS-HrpR interactions, HrpV binding to HrpS may induce conformational changes at the surface on HrpS that can both interact with HrpS and HrpR. Findings suggest that *in trans* regulation by HrpV of the heteromeric HrpRS bEBP is distinct from the *in trans* regulation of the homomeric PspF by PspA since: i) HrpV is likely to bind to a different subunit surface on the AAA+ domain compared with PspA (Zhang *et a*l., 2013), ii) HrpV appears to only directly interact with a subset of subunits (HrpS) in the

likely active hexameric ring assembly of bEBPs (Joly & Buck, 2010) whereas PspA binds to PspF in a 6:6 ratio.

According to our data two potential organizations of HrpRS hexamer assemblies are: i) S_1, S_2, R_3, S_4, S_5, R_6, ii) R_1, S_2, S_3, S_4, S_5, R_6. They reflect HrpS self-association and lack of direct HrpR self-association. In ii) subunits R_1 and R_6 would favour over i) an open ring structure as proposed for PspF and NtrC (Joly & Buck, 2011; Sysoeva *et al*., 2013).

Supplementary Fig. 2

Transcription activity *in vivo* of HrpS variants, within HrpRS complexes, that escapes negative regulation by HrpV. WT HrpRS proteins or HrpRS variants (HrpS variants with alanine substitutions- S26A, R90A, R185A and H251A or original mutations- I26S, R90H, R185H and H251Q) were co-expressed from pAPT110 vector; HrpV was expressed from pBAD vector. Negative controls, pAPT110 (empty vector) or pAPT110 with pBAD*hrpV*. Reporter strain MJ2806 has a chromosomal *hrpL::lacZ* fusion and HrpRS and HrpV constructs were grown in LB liquid culture supplemented with 25µg/ml Kan and 30 µg/ml Cm, O/N culture was diluted 50 times, grown at 25°C until OD_{600} 0.4-0.6, induced with 0.5 mM IPTG (HrpRS) and 0.2% arabinose (HrpV) and grown for a further three hours. Activity of *hrpL::lacZ* promoter fusion was measured using β-Galactosidase assay. Each bar represents the mean value with standard deviations of results obtained from three independent biological samples.

HrpS(R90H) is located at the end of Loop1. In PspF (Joly *et al.*, 2010) this mutation had no effect on ATPase activity, it moderately decreased interactions with the closed promoter complex, and had drastic negative effect on remodelling the closed promoter complex and likely contributes to controlling Loop1 exposure for binding to σ^{54}.

HrpS(R185H) is located between R fingers and sensor II. In bEBP DctD mutations in this region could affect relative orientation of the Sensor II helix or interfere with oligomerization of the protein.

HrpS(H251Q) is located at the C-terminal end of the AAA+ domain.

Supplementary Fig. 3

a) Binding interactions between the HrpS variants I26, R90, R185 and H251fused to T18 AC fragment and assayed in the presence of: a) HrpV fused to T25 AC fragment, b) HrpR fused to T25 AC fragment, c) HrpS fused to T25 AC fragment. Interactions between the hybrid proteins were measured and quantified as described in Fig.3 legend. The positive control with constructs expressing T18-zip and T25-zip yielded 2557±589, while negative control with empty vectors pUT18C and pKT25 yielded 42±9. Each bar represents the mean value with standard deviations of results obtained from three independent biological samples.

Supplementary Fig. 4

Western blotting of the Hrp proteins expressed in the BACTH system recognised by corresponding antibody. Arrow shows the protein band.
